# Pattern of neural responses to verbal fluency shows diagnostic specificity for schizophrenia and bipolar disorder

**DOI:** 10.1186/1471-244X-11-18

**Published:** 2011-01-28

**Authors:** Sergi G Costafreda, Cynthia HY Fu, Marco Picchioni, Timothea Toulopoulou, Colm McDonald, Eugenia Kravariti, Muriel Walshe, Diana Prata, Robin M Murray, Philip K McGuire

**Affiliations:** 1 Institute of Psychiatry, King's College London, De Crespigny Park, London UK

## Abstract

**Background:**

Impairments in executive function and language processing are characteristic of both schizophrenia and bipolar disorder. Their functional neuroanatomy demonstrate features that are shared as well as specific to each disorder. Determining the distinct pattern of neural responses in schizophrenia and bipolar disorder may provide biomarkers for their diagnoses.

**Methods:**

104 participants underwent functional magnetic resonance imaging (fMRI) scans while performing a phonological verbal fluency task. Subjects were 32 patients with schizophrenia in remission, 32 patients with bipolar disorder in an euthymic state, and 40 healthy volunteers. Neural responses to verbal fluency were examined in each group, and the diagnostic potential of the pattern of the neural responses was assessed with machine learning analysis.

**Results:**

During the verbal fluency task, both patient groups showed increased activation in the anterior cingulate, left dorsolateral prefrontal cortex and right putamen as compared to healthy controls, as well as reduced deactivation of precuneus and posterior cingulate. The magnitude of activation was greatest in patients with schizophrenia, followed by patients with bipolar disorder and then healthy individuals. Additional recruitment in the right inferior frontal and right dorsolateral prefrontal cortices was observed in schizophrenia relative to both bipolar disorder and healthy subjects. The pattern of neural responses correctly identified individual patients with schizophrenia with an accuracy of 92%, and those with bipolar disorder with an accuracy of 79% in which mis-classification was typically of bipolar subjects as healthy controls.

**Conclusions:**

In summary, both schizophrenia and bipolar disorder are associated with altered function in prefrontal, striatal and default mode networks, but the magnitude of this dysfunction is particularly marked in schizophrenia. The pattern of response to verbal fluency is highly diagnostic for schizophrenia and distinct from bipolar disorder. Pattern classification of functional MRI measurements of language processing is a potential diagnostic marker of schizophrenia.

## Background

Impairments in language and executive function are a key feature of schizophrenia [1]. Deficits have also been observed in bipolar disorder, although these may be less pronounced [2]. Such performance deficits may be the effect of a common mechanism that is shared by both illnesses or they may reflect abnormalities specific to each disorder [3-5]. A common mechanism would be consistent with a dimensional approach to cognitive deficits in psychotic disorders [6]. However, neural features that are specific to each disorder may distinguish the substantive clinical and prognostic differences that exist between schizophrenia and bipolar disorder [7] and lead to the development of diagnostic biomarkers [8].

Phonological verbal fluency requires the generation of words from a letter cue [9]. This task places high requirements on executive function [10] and is thus dependent on performance in the prefrontal cortex, in particular the dorsolateral prefrontal cortex [11]. In healthy individuals, verbal fluency is associated with a network of activation in cortical and subcortical regions [9,12]. However, significant functional abnormalities are revealed in schizophrenia [13] and in bipolar disorder [14].

In the present study, we used the verbal fluency task to investigate the functional neuroanatomy of executive function in schizophrenia and bipolar disorder. We recruited a large sample of patients with schizophrenia and bipolar disorder and matched healthy controls. In order to avoid possible confounding effects of active symptomatology [13,15], only patients who were in clinical remission were included. All subjects underwent functional magnetic resonance imaging (fMRI) while performing a verbal fluency task [9,13]. As task performance also modulates brain activation differences [13,16], we matched the groups on their performance in the verbal fluency task during the fMRI scan. We examined regional activity in the dorsolateral prefrontal cortex [3,4] and potential selective dysfunction in other frontal [3,5] and non-frontal [3,4] areas. We also applied a machine learning analysis [8,17] to test the hypothesis that the pattern of regional brain responses would correctly identify the diagnosis for each participant at the individual level.

## Methods

### Participants

All subjects were English-speaking, medically healthy and right-handed. Patients with schizophrenia or bipolar disorder were diagnosed with DSM-IV criteria [18] by consultant psychiatrists from clinical interviews, medical chart review, and consultation with patients' psychiatrists. All patients with schizophrenia were in remission as assessed by Scale for the Assessment of Positive Symptoms [19] (SAPS) and the Scale for the Assessment of Negative Symptoms [20] (SANS). All patients with bipolar disorder were of Type I bipolar disorder, in an euthymic state, as assessed by the Beck Depression Inventory [21], Hamilton Depression Rating Scale [22], Altman Self-Rating Mania Scale [23], Young Mania Rating Scale [24]. Exclusion criteria were a co-morbid psychiatric or neurological disorder in patient groups, including substance abuse or dependence within the previous 6 months or a history of a psychiatric or neurological disorder in healthy volunteers. All participants provided written, informed consent with approval from the South London and Maudsley (SLAM) NHS Trust (Research) ethics committee. There were a total of 104 subjects: 32 patients with schizophrenia in remission, 32 bipolar disorder in an euthymic state, and 40 healthy controls (Table 1). Subject MRI scans were acquired from fMRI studies conducted at the Institute of Psychiatry, SLaM NHS Trust. Data were obtained from 4 studies: 1) verbal fluency study of schizophrenia and healthy controls [9,13]; 2) Maudsley Family study, patients with schizophrenia or bipolar disorder and their family members [25]; 3) Maudsley Schizophrenia Twin study; and 4) Maudsley Bipolar Twin study, which involved twin pairs concordant and discordant for schizophrenia and bipolar disorder, respectively, and healthy control twins [26]. From the Family study samples, 1 subject was randomly selected from each family, and from the Twin studies, only 1 subject from each twin set was included to ensure that each individual could be considered statistically independent from the other subjects in the final sample; the inclusion of non-independent subjects could have reduced the variance within each of the groups thereby increasing separation between diagnoses artificially. Groups were matched by their performance on the verbal fluency task in the number of correctly produced words during the fMRI scan. The medication status of the patients with schizophrenia consisted of 20 patients taking atypical antipsychotics, 10 conventional antipsychotics, and 2 were not receiving any medication. The mean chlorpromazine equivalent dosage was 625.9 mg daily (SD = 411.2 mg). The mean SAPS rating was 9.52 (SD = 8.85) and SANS rating was 8.31 (SD = 4.96), reflecting their clinical status as being in remission. In the bipolar patient group, 26 patients were receiving medications and 6 patients were medication-free: 24 with mood stabilizer medication, which was lithium in 14 cases (mean dosage of 817.86 mg daily (SD = 207.91 mg); 8 were also taking regular doses of antipsychotic medication; and 8 subjects antidepressants. From the Maudsley Family study, the 16 bipolar patients had a Beck Depression Inventory mean of 7.76 (SD = 7.16) and a Altman Self-Rating Mania Scale mean of 3.65 (SD = 2.69). From the Maudsley Bipolar Twin study, the clinical ratings were a mean of 5.44 (SD = 8.61) in the Hamilton Depression Rating Scale and mean of 2.00 (SD = 3.71) in the Young Mania Rating Scale. All of the bipolar patients were in a euthymic state, none fulfilled criteria for a major depressive or manic episode or had any active psychotic symptoms.

**Table 1:** Demographic and clinical characteristics

	Healthy Controls	Bipolar Disorder	Schizophrenia	p-value
Number of subjects	40	32	32	
Men:Women	20:20	14:18	26:6	0.005
Twins:Non-twins	21:19	16:16	15:17	0.90
Caucasian:Non-caucasian	36:4	30:2	26:6	0.27
Age	35.8 (11.3)	41.4 (11.9)	35.5 (10.7)	0.09
Years of education	14.7 (2.7)	15.4 (2.8)	13.7 (2.6)	0.16
IQ	110.6 (13.4)	110.2 (12.5)	105.4 (11.1)	0.19
Disease duration		16.9 (12.3)	11.4 (7.3)	0.29
Performance in fMRI task				
Errors, easy condition	3.5 (3.1)	4.5 (4.9)	5.2 (3.7)	0.20
Errors, hard condition	6.8 (5.1)	6.3 (6.1)	8.5 (4.2)	0.22

Mean values are presented with the standard deviation in parenthesis. Age and disease duration are presented in years. Performance during the fMRI verbal fluency task was matched for the groups, and the number of errors is presented for the easy and hard conditions of the task.

### Verbal Fluency Task

The experimental condition was a phonological letter fluency task [10] with 2 levels of difficulty [9]. Subjects were instructed to overtly generate a word in response to a visually presented letter shown at a rate of one every 4 seconds, while avoiding proper names, repetitions and grammatical variations of previous words [10]. If subjects were unable to think of a response, they were asked to say "pass". The difficulty of the condition depended on which set of letters was presented. The letters were categorized as "easy" and "difficult" according to the mean number of erroneous responses subjects generated in a previous study [9]. There were 7 presentations of each letter within a 28 seconds experimental block, followed by the control condition which was repetition of the word "rest" presented at the same rate (28 seconds control block). The "easy" set of letters were: T, L, B, R, S or T, C, B, P, S; and the "difficult" set of letters were: O, A, N, E, G or I, F, N, E, G. The order of presentation was randomized between subjects. Verbal responses during scanning were recorded.

### Data Acquisition

All MRI scans were acquired following the same procedure with the same acquisition system [9,13], which is regularly monitored to ensure the quality and stability of fMRI measurements [27]. Seventy-four T2*-weighted gradient-echo single-shot echo-planar images were acquired on a 1.5-T, neuro-optimized IGE LX System (General Electric, Milwaukee) at the Maudsley Hospital, SLAM NHS Trust. Twelve noncontiguous axial planes (7 mm thickness, slice skip 1 mm) parallel to the anterior commissure-posterior commissure line were collected over 1100 msec in a clustered acquisition sequence, in order to allow subjects to make overt responses in relative silence (TE = 40 msec, flip angle = 70 degrees). A letter was presented (remaining visible for 750 msec, height: 7 cm, subtending a 0.4 degrees field-of-view) immediately after each acquisition, and a single overt verbal response was made during the remaining silent portion (entire duration = 2900 msec) of each repetition (TR = 4000 msec).

### fMRI Data Analysis

The fMRI data were analyzed using SPM5 (Wellcome Department of Imaging Neuroscience, London, UK). MRI scans were realigned to remove motion effects, transformed into standard MNI space, and smoothed with an isotropic Gaussian filter (FWHM = 8 mm). A mask was applied to select intra-cerebral voxels, and the data were high-pass filtered (cutoff 128 sec) to remove low-frequency drifts.

Subject-level model estimation was performed by convolving a canonical hemodynamic response function model on correct and incorrect trials separately. Realignment parameters were included as nuisance covariates in the General Linear Model (GLM) to adjust for residual motion. For each subject, statistical images were computed representing the contrast word production (correct trials only) minus baseline for easy and difficult letter trials. These subject-level images were included in a second-level random effects ANOVA (analysis of variance) which modeled the diagnostic group effect (schizophrenia, bipolar and control) and included task difficulty as intra-subject factor and gender, age and antipsychotic dosage (chlorpromazine equivalent) as potential confounding factors. As heterogeneous mood stabilizer drugs cannot be easily converted into a single equivalent value we did not devise an adjustment strategy for these drugs. Inferences on the model were conducted using a height threshold of *p *< 0.001 (uncorrected), followed by a corrected cluster-level significance level of *p *< 0.05, corrected for multiple comparisons. For those clusters of activation showing a significant main effect of diagnostic group, an exploratory post-hoc analysis was conducted using analogous repeated-measures ANOVA models on the cluster peaks of activation to explore the direction of the group differences, by extracting the beta estimate of activation at the voxel of peak activation for each cluster.

### Machine learning classification analysis

We additionally conducted a pattern classification analysis to investigate whether clinical diagnosis could be determined on the basis of activation patterns alone. We employed Support Vector Machines (SVM) classification analysis [28], which has been shown to be a powerful tool for statistical pattern recognition. SVM has proven to be a robust and versatile approach for clinical prediction, as demonstrated by its consistently high performance in head-to-head methodological comparisons of diverse machine learning methods performed with fMRI data [29] and other high-dimensional clinical datasets such as proteomics [30] and genomics [31]. Our group has also demonstrated the potential of linear SVM for neuroimaging-based prediction in depression [8,17]. The inputs to the SVM classification analysis were the activation patterns of each participant during easy and difficult verbal fluency, thresholded using the ANOVA test for group differences. These activation patterns were then fed to a multi-class linear SVM classifier [32] that learned the statistical boundaries that best separates the groups. Afterwards, this boundary can be used to obtain a diagnostic prediction for the scan of an undiagnosed subject. As implemented here, the procedure finds the boundary that maximises the expected overall classification accuracy in new, unclassified examples. This boundary therefore treats as equivalent two types of errors: false positives (FP, e.g. labelling a control as patient) and false negatives (FN, misdiagnosing a patient as a control). For some clinical applications, such types of errors may not be equivalent. For example, if the clinical goal is to confirm the presence of a disorder, a better classification rule would be one that ensures a low FP rate (high specificity) while tolerating a higher FN rate (lower sensitivity) and potentially a lower overall classification accuracy. Our purpose in the present paper, though, was to establish the potential of the neural correlates of verbal fluency as a diagnostic biomarker, and this proof-of-principle goal benefits from optimising the overall diagnostic accuracy rather than sensitivity or specificity.

To avoid circularity, i.e. using the same data to create a classification rule and test its performance, which can lead to over-optimistic results in diagnostic studies, we employed leave-one-out cross validation (LOOCV). LOOCV entails training the model (fitting both the second-level ANOVA and the linear SVM model) with all subjects minus one, and using the remaining single individual to test the accuracy of the prediction. This process is iterated until the sample is exhausted. We used permutation testing to determine the overal model performance, that is whether the observed performance for the diagnostic classification of bipolar and schizophrenia subjects could have been expected by chance alone, by repeating the whole ANOVA model estimation and linear SVM classification process 1000 times after successive random permutation of the diagnostic labels of subjects. The p-value of the experimental accuracies was computed using the resulting null-hypothesis distributions. Because of the gender imbalance present in our sample, we also repeated this classification procedure for male subjects alone. The cost parameter C of the SVM model was optimized through cross-validation within each training sample. Additional analyses were performed using the following packages of the R statistical software [33]: AnalyzeFMRI which offers input/output, visualisation and analysis functions for fMRI data and the e1071 package, which supplies an interface to the libsvm library http://www.csie.ntu.edu.tw/~cjlin/libsvm/. Coordinates are reported in MNI space.

## Results

There were no significant differences in the demographic features of the groups in IQ, years of education, ethnicity, disease duration, percentage of twins in each group, or performance in the fMRI verbal fluency task (Table 1). There was a higher proportion of male subjects with schizophrenia than in other groups.

### Conventional activation group analysis

**Figure 1:**
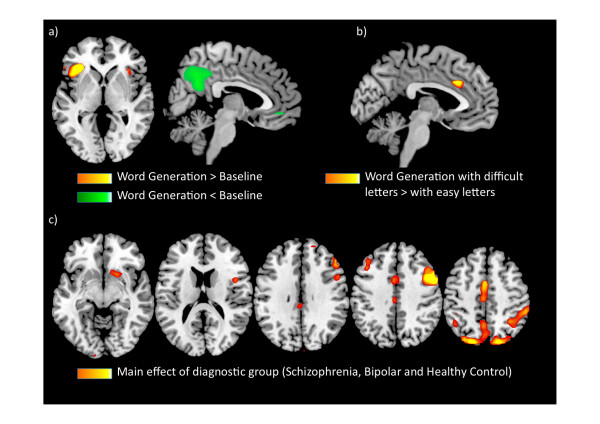
**Patterns of activation during word generation**. Significant activations during verbal fluency according to SPM random-effects analysis for the whole subject sample (a and b, slices at x = 0, z = +4 and x = -4) and diagnostic effects (c, slices at z = -8,16,40,48), adjusted by sex and antipsychotic dosage. (MNI space, images are in MNI space and +x on the right). Results are multiple-comparisons corrected with cluster-level significance level of *p *< 0.05.

The main effect of verbal fluency revealed activation in a distributed network of regions that is well associated with word production [12], encompassing the bilateral inferior frontal and insular cortices, left superior temporal cortex, thalamus, and the dorsal anterior cingulate cortex which showed a greater response for the more difficult letters. Verbal fluency was also associated with less activity in the precuneus and rostral anterior cingulate gyrus compared to word repetition (Figure 1, Table 2). There was no significant effect of antipsychotic medication dosage on regional brain activity.

**Table 2:** Significant effects of word generation, task difficulty and group effects during the performance of a verbal fluency task by subjects with schizophrenia, bipolar disorder and healthy controls.

		Coordinates			
	Brodmann area	x	y	z	Z max
Word generation > repetition					
Inferior frontal gyrus	BA47	-34	28	0	4.58
	BA45	-50	28	16	3.89
	BA44	-52	12	24	5.44
Inferior frontal gyrus/insula	BA47	36	24	0	3.5
Inferior frontal gyrus/orbital	BA47	-50	36	-8	2.95
Superior temporal gyrus	BA 38	-52	20	-16	4.18
Thalamus		-8	-14	10	3.37
					
Word generation < repetition					
Precuneus	BA 7	-2	-68	48	4.8
Ventral anterior cingulate	BA10/25	4	44	-8	4.11
					
Word generation with difficult > easy letters					
Dorsal anterior cingulate (rostral, supracallosal)	BA24/32	-4	24	32	2.65
					
Effect of diagnostic group					
Schiz. > bipolar > control					
Dorsal anterior cingulate (caudal, supracallosal)	BA24	-2	0	40	3.12
Middle frontal gyrus	BA46/44	-40	32	40	3.81
Putamen		18	14	-8	3.23
Schiz. > (bipolar, control)					
Inferior and middle frontal gyrus	BA44/9/6	44	12	40	4.29
Superior frontal gyrus	BA9	18	52	32	3.18
Inferior frontal gyrus	BA44/6	48	10	16	2.75
(Schiz., bipolar) > control					
Precuneus	BA7	-4	-66	48	4.24
Precuneus/Superior occipital cortex	BA7	18	-82	48	4.12
Angular/supramarginal gyrus	BA39/40	56	-38	48	3.29
Angular gyrus	BA39	-42	-58	48	2.72
Posterior cingulated	BA23	0	-30	32	2.83

**Figure 2:**
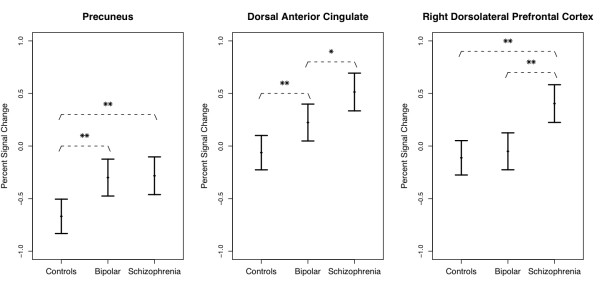
**Group differences in activation in selected areas**. Mean percent change of the BOLD signal in selected areas, with 95% confidence intervals. The locations are precuneus (cluster peak coordinates x = -4, y = -66, z = 48, Brodmann area 7) where bipolar and schizophrenia patients demonstrated reduced deactivation relative to healthy controls, dorsal anterior cingulate (x = -2, y = 0, z = 40, BA24), where both patient groups showed increased activation and right dorsolateral prefrontal cortex (x = 44, y = 12, z = 40, BA44/9), where the activation was higher only for schizophrenia patients. One asterisk denotes that differences are significant at *p *< 0.01, two asterisks denotes *p *< 0.001.

The main effect of group was evident in the anterior cingulate, dorsolateral prefrontal, and inferior frontal regions, and in the putamen (Figure 2, Table 2). Patient with schizophrenia showed the greatest activity in the dorsal anterior cingulate, left dorsolateral prefrontal cortex and right putamen, followed by patients with bipolar disorder and then healthy controls. In the right inferior frontal and dorsolateral prefrontal cortex, patients with schizophrenia showed greater activation than both patients with bipolar disorder and healthy controls. Both patient groups showed greater activity in the precuneus, posterior cingulate and angular gyrus bilaterally relative to healthy controls, reflecting relatively reduced deactivation. There were no areas in which healthy controls showed more activation than either patient group.

### Machine learning classification analysis

The classification analysis based on the patterns of brain activation to verbal fluency correctly identified individuals with schizophrenia at an accuracy of 92% (sensitivity = 91%, specificity = 92%, the probability of achieving such classification performance by chance is *p *< 0.001). The accuracy of classification for individuals with bipolar disorder was lower at 79% (sensitivity = 56%, specificity = 89%, *p *< 0.001); 14 of the 32 bipolar subjects were misclassified, 12 of them as healthy controls. As there were a significantly greater proportion of male subjects in the schizophrenia group, we also repeated the classification analysis after restricting the sample to the male subjects only. In the male subjects, the classification results were similar as the accuracy for schizophrenia was 87% (sensitivity = 88%, specificity = 85%, *p *< 0.001) and for bipolar disorder was 73% (sensitivity = 57%, specificity = 91%, *p *< 0.001).

## Discussion

### Group differences in activation

Regional brain responses to the verbal fluency task demonstrated significant areas of abnormal shared circuitry as well as distinct functional differences in schizophrenia and bipolar disorder. The verbal fluency task engaged language production regions [12] as well as deactivations within the default-mode network [34].

Both patient groups showed increased activation in the left dorsolateral prefrontal cortex, while patients with schizophrenia engaged the right inferior frontal and right dorsolateral prefrontal regions more strongly than both bipolar disorder and healthy participants. The lateral prefrontal cortex has a central role in executive control and response selection, in the dynamic allocation of attentional resources, and in filtering out unwanted stimuli [35]. The right inferior frontal cortex in particular has been linked to the inhibition of inappropriate responses [35]. These components of executive control contribute to maintaining task performance during verbal fluency. In healthy subjects, executive control in lateral prefrontal cortex is modulated by dorsal anterior cingulate activity during performance monitoring [36]. The dorsal anterior cingulate demonstrated increased task-related recruitment in patients relative to healthy controls, with schizophrenia subjects showing the greatest activation relative to bipolar and healthy control subjects.

Cytological, structural and functional abnormalities in the anterior cingulate cortex have been identified in both schizophrenia [37] and bipolar disorder [38]. In particular, dorsal anterior cingulate hyperactivation during executive processing has been reported in schizophrenia [39] and bipolar disorder [40]. Dorsal anterior cingulate activity is linked with online task monitoring, which may contribute to maintaining normal task performance in patient populations [41]. Moreover, the increased engagement of the left dorsolateral prefrontal cortex in both groups of patients in the present study may be secondary to the increased response in dorsal anterior cingulate. Our findings are also congruent with the evidence of greater morphological changes in frontal areas in schizophrenia [42] than bipolar disorder [38].

Both patients with schizophrenia and bipolar disorder showed a relative failure to deactivate the precuneus, posterior cingulate and angular gyri as compared to healthy controls, which is consistent with overactivity of the default-mode network during task performance [34]. A similar pattern of deactivations has previously described during working memory [43-45] and attentional tasks [46,47] in schizophrenia as well as other psychiatric disorders [48]. Reduced deactivation of the default-mode network has been linked to lapses of attention [49,50] and predicts task error [51] in healthy individuals, suggesting that default-mode network overactivity in patient populations may contribute to error proneness and performance deficits. The present findings extend this abnormality to a task involving language and executive functions in both schizophrenia and bipolar disorder.

We also found a similar degree of overactivation in the putamen in both schizophrenia and bipolar subjects. The striatum has reciprocal connections to both the anterior and posterior cingulate cortices [52], and is involved in executive processing tasks [53]. Polli and colleagues [54] observed a negative correlation between error rate and anterior cingulate and putamen activation during an antisaccade paradigm in both schizophrenia and healthy controls. The exaggerated putamen response in the patient groups may reflect a hyperactive response monitoring system or perhaps a relative failure to use more automated strategies for task implementation [55].

The greatest differences in activation during verbal fluency were evident between schizophrenia patients and healthy controls, with bipolar subjects occupying the middle ground. Two recent studies contrasted regional brain responses to executive processing using visual memory [4] and semantic language production [3] in the these disorders. While diagnostic effects were also identified in dorsal prefrontal and inferior frontal cortex, there were additional task-specific differences in the ventral striatum, orbitofrontal [3] and visual cortices [4]. The direction of the differences also varied according to the task, with bipolar subjects revealing a similar intermediate pattern of anomalies between healthy controls and schizophrenia in the visual working memory task [4], which is consistent with our findings.

### Diagnostic classification analysis

The classification analysis revealed over 90% sensitivity and specificity for the detection of schizophrenia relative to both bipolar subjects and matched healthy controls. Similarly high diagnostic utility has been reported for the diagnosis of schizophrenia based on the fMRI neural correlates of an auditory oddball task [56], and VBM-derived structural differences [57,58]. Notably the basis for such accurate diagnostic decision has not been identical across studies and tasks: for instance, while prefrontal deficits were prominent in both VBM-based and fMRI-based classification studies, abnormalities in posterior regions such as precuneus and posterior cingulate have only been reported in fMRI-based classification [[56], and the present paper]. Our work on neuroimaging-based prediction in depression has also shown that functional and structural MRI may convey complementary predictive information [8,17,59]. A promising way to further optimize diagnostic performance may therefore be the fusion of complementary information from structural and functional MRI that may be superior to either of them in isolation. Increased performance, even above the encouraging figures reported so far, is likely to be necessary to achieve clinical utility.

In the classification analysis, the pattern of activation generated higher diagnostic sensitivity for schizophrenia than bipolar disorder. This discrepancy in diagnostic potential between the disorders may be linked to the existence of specific abnormalities associated with schizophrenia in right frontal regions, whereas no such anomalies were apparent in bipolar disorder. Also using a classification approach, Calhoun and colleagues [56] achieved high diagnostic accuracy in classifying bipolar and schizophrenia subjects using temporal and default-mode network activity during an auditory oddball task. Similar to our findings, the majority of patients with schizophrenia were correctly identified. However, their classification of bipolar subjects was more accurate with a sensitivity of 83% perhaps due to active psychotic symptoms present in almost a third of the patients with bipolar disorder, while the present study only included bipolar patients in an euthymic state without any psychotic symptoms. Our findings suggest that tasks with prominent executive and attentional subcomponents may be more discriminative for schizophrenia than for bipolar disorder.

An observation in both Calhoun and colleagues [56] and the present work, is the relevance of default mode network abnormalities for diagnostic purposes. We had anticipated that functional differences would be largely confined to prefrontal regions. This convergence of findings across two different tasks suggests that applying machine learning classification to resting state data may also be a promising line of enquiry.

### Limitations

A limitation of the present study was the medication status of the patients. Although we did not find any significant effects of antipsychotic drug dose in our sample, there is some evidence of modulatory effects of psychoactive drugs on brain activation as antipsychotic and lithium treatment affect frontal activation [60,61] and antipsychotic medication has been linked to functional and structural changes, particularly in prefrontal areas and the striatum [61-63]. If present, such confounding may result in increased brain function differences between patients and controls, and also between schizophrenia and bipolar patients, as the latter are less likely to require long-term antipsychotic treatment. For classification, this medication effect could result in increased separation between groups and therefore increased classification accuracy than would be the case in unmedicated samples. Replication of our findings in patients who are medication-free is thus necessary to exclude these potentially confounding effects, particularly as any diagnostic tool would be most useful prior to the initiation of medication. It is worth pointing out, however, that our findings are similar to those demonstrated in medication-free samples in which medication naïve subjects with prodromal symptoms showed increased right prefrontal activation during verbal fluency [64], unaffected first-degree relatives of patients with schizophrenia demonstrated increased recruitment of the default-mode network [44], dorsolateral prefrontal cortex [65] and right inferior frontal gyrus [5] during executive processing tasks, and children with subclinical psychotic symptoms showed dorsal anterior cingulate hyperactivation in response inhibition tasks [66]. This convergence of results between our findings and those of studies in drug-free subjects suggests that our classification findings may be generalizable to unmedicated patients.

Another limitation is that the pattern of activation in the patient groups could have been influenced by differences in active psychopathology and past clinical symptoms as prefrontal activation may be modulated by negative and disorganization symptoms in schizophrenia [15] and by the affective state in bipolar disorder [67]. It is also possible that past psychotic symptoms in bipolar subjects may have impaired their differentiation from schizophrenia subjects. While we can confirm that all bipolar subjects were euthymic and none were actively psychotic at the time of the scan, the presence of psychotic symptoms in past manic or depressive episodes was not consistently recorded during the assessment.

Bipolar subjects were also on average 6 years older than either of the other two groups, which may have facilitated diagnostic classification. Patient diagnoses were ascertained through consensus methods by consultant psychiatrists, rather than with a structured diagnostic interview, potentially leading to lower diagnostic certainty. Finally, although we used leave-one-out cross-validation to ensure that the classification algorithm was tested in different subjects from the ones on which it was developed, a complete assessment of the clinical utility of the diagnostic algorithm should include testing in a fully independent set of patients, recruited in a different clinical setting.

## Conclusions

In summary, significant functional abnormalities were evident in the neural responses to verbal fluency in both schizophrenia and bipolar disorder. The impairments were most marked in schizophrenia, while patients with bipolar disorder showed an intermediate degree of response relative to schizophrenia and healthy controls. The pattern of brain activity showed high diagnostic sensitivity for schizophrenia, but reduced accuracy in identifying bipolar disorder as these patients were often *mis*classified as healthy controls. The functional neuroanatomy of verbal fluency shows strong potential as a diagnostic marker for schizophrenia which is distinct from bipolar disorder.

## Abbreviations

fMRI: Functional Magnetic Resonance Imaging; SVM: Support Vector Machines.

## Competing interests

The authors declare that they have no competing interests.

## Authors' contributions

CHYF, MP, TT, CM, MW, RMM and PKM were involved in the design of the original studies, and SGC, CHYF conceived the present analysis. CHYF, MP, TT, CMD, EK, MW were involved in data collection, which was supervised by RMM and PKM. SGC, CHYF, MW, DP have been involved in data management and analysis. SGC and CHYF prepared the first draft of the manuscript, and all authors read and have been involved in giving comments on this paper.

## Pre-publication history

The pre-publication history for this paper can be accessed here:

http://www.biomedcentral.com/1471-244X/11/18/prepub
